# Deubiquitylation and stabilization of ARMC5 by ubiquitin‐specific processing protease 7 (USP7) are critical for RCC proliferation

**DOI:** 10.1111/jcmm.16306

**Published:** 2021-02-05

**Authors:** Guobei Yan, Na Liu, Jun Tian, Yanli Fu, Wei Wei, Jingjing Zou, Suping Li, Qing Wang, Kai Li, Junhua Wang

**Affiliations:** ^1^ Department of Laboratory The Second People's Hospital of Jiaozuo the First Affiliated Hospital of Henan Polytechnic University Jiaozuo China; ^2^ Henan Polytechnic University Jiaozuo China; ^3^ Department of Laboratory Jiaozuo Maternal and Children’s Hospital Jiaozuo China

**Keywords:** ARMC5, proliferation, renal cell carcinoma, USP7

## Abstract

The ubiquitin‐proteasome system is an essential regulator of ARMC5, which serves as a new tumour suppressor protein for inhibiting meningiomas and hereditary adrenocortical tumorigenesis. However, the precise mechanism for the deubiquitination of ARMC5 is still not fully understood. A Western blot analysis of ARMC5 was performed and showed that the expression of ARMC5 was decreased in the renal cancer cell tissues and lines. By screening a deubiquitinase library, we identified USP7 as a potential ARMC5 associated deubiquitinase. In this paper, we demonstrated that there was an interaction between USP7 and ARMC5 in vivo and in vitro. Employing the overexpression and knockdown assay indicated that USP7 could greatly increase the steady state of ARMC5 through the ubiquitin‐proteasome pathway and regulate ARMC5 ubiquitination. Moreover, USP7 altered cell cycle G1/S phases and regulated renal cancer cell proliferation by targeting ARMC5. Together, these results suggest that USP7 plays an important role in the RCC proliferation through modulating ARMC5 stability.

## 1 INTRODUCTION

Renal cell carcinoma (RCC), which is the primary malignant tumour of the kidneys in adults, generally shows asymptomatic in the early stage.^1^ Early‐stage RCC could be treated by radical nephrectormy in clinical practice.^2^ However, there was no satisfactory treatment yet for both chemotherapy‐ and radiotherapy‐resistant RCC in the clinic.^3^ Like most tumours, abnormal cell cycle and longer survival are involved in RCC tumorigenesis.^4^, ^5^ Therefore, it is essential to explore the molecular mechanisms of RCC proliferation, which will provide important insight into new therapeutic approaches to improve the prognosis of RCC patients.

Primary macronodular adrenocortical hyperplasia (PMAH) is a disease, which is characterized by bilateral adrenal cortical nodules clinically.^6^ It has previously been reported that PMAH may include the entire process from hyperplasia to tumour formation, representing the transitional from hyperplasia to carcinoma.^7^ An analysis of familial clustering PMAH patients revealed that the differentially expressed genes are also closely related to tumorigenesis.^8^ Meantime, the current researches show that ARMC5 gene mutations have been deemed to the main cause of PMAH,^9^, ^10^ within ARMC5 mutation occurs in 50% PMAH patients.^11^ These mutations have been recently identified at the germline and somatic levels, and most of the ARMC5 mutations were meaningless (12/54, 22%) and frameshifted (21/54, 38%), resulting in significant loss of function. These data indicate that ARMC5 may be a tumour suppressor gene. Moreover, the epidemiological findings there is a relation with meningioma and ARMC5 mutation.^12^ In vitro experiments show ARMC5 missense variants identified in human adrenocortical cancer cell line (H295R), leads to loss of apoptosis activity,^13^ suggesting that these variants affect ARMC5’s pro‐apoptotic function. Together, the above researches show that ARMC5 defects have a pathogenic role in diseases and ARMC5 defects may conduce to tumorigenesis. However, it remains unclear that the expression and significance of ARMC5 in RCC and whether it is involved in RCC progression.

Importantly, deubiquitination and ubiquitination are the two main types of protein post‐translational modification that can regulate protein homeostasis in biological cells.^14^, ^15^ It has long been known that abnormal deubiquitination or ubiquitination often results in disease, especially in cancer progression.^16^, ^17^, ^18^ Over hundred known DUBs regulate a wide variety of cellular events^19^, ^20^ and reverse these complex ubiquitinated proteins.^21^, ^22^, ^23^ These deubiquitinases are divided into 6 subfamilies, which consist of different cysteine and metallopeptidases.^24^ Ubiquitin‐specific processing protease 7 (USP7), belongs to cysteine protease, serves as a member of the deubiquitinating enzyme.^25^ USP7, as one of the well‐known tumour‐associated DUBs, is to participate in the regulation of stability and functions of cellular proteins, including p53, PTEN^26^ and involve in the TGFβ^27^ and NF‐κB^28^ signalling pathways. Meanwhile, USP7 plays an important role in the development and progression of diseases.^29^ However, the expression levels and clinical significance of USP7 in RCC are not well understood, and it remains unclear whether it is involved in RCC proliferation, apoptosis and migration progression.

In the study, we demonstrated that ARMC5 was decreased at protein levels in the renal cancer cell tissues and lines. We identified USP7 as an ARMC5 associated deubiquitinase by screening the library. Meantime, there was an interaction between USP7 and ARMC5 in vivo and in vitro directly. We confirmed USP7 as the first deubiquitylase that stabilized protein ARMC5 by inhibiting the degradation of ARMC5. Silencing of USP7 enhanced ARMC5 ubiquitination and accelerated cell cycle G1/S, subsequently promoted RCC cell proliferation. Together, we clarify that USP7 plays an important role in the regulation of RCC proliferation through modulating ARMC5 stability.

## 2 MATERIALS AND METHODS

### 2.1 Reagents, tissue specimens and cell culture

The primary and secondary antibodies were purchased from Abcam and Santa Cruz Biotechnology. All experiments were performed in accordance with the Guidelines of the First Affiliated Hospital of Henan Polytechnic University (Jiaozuo, China), and experiments were approved by the ethics committee at the First Affiliated Hospital of Henan Polytechnic University. Informed consents were obtained from human participants of this study. The renal carcinoma patients’ and adjacent normal tissues frozen section were, respectively, collected from the Pathology Department, the Second People's Hospital of Jiaozuo City.

786‐O and 769‐P (Procell) were cultured in RPMI 1640 (Gibco, catalog no. 12633012) supplemented with 10% FBS (Cat#1500‐500, Seradigm). A498, ACHN and HEK293T cells (Procell) were cultured in DMEM (Gibco, catalog no. 11965092) supplemented with 10% FBS. Cells were incubated at 37°C in 5% CO_2_/95% mixed ambient air.

### 2.2 Western blot analysis and co‐immunoprecipitation (co‐IP) assay

Briefly, the processed cells were incubated in RIPA buffer (Thermo Fisher Scientific), and then, the centrifuged protein lysates were separated employing the SDS/PAGE. Separated proteins were transferred to a immobilon‐PVDF Transfer Membrane (Millipore), which was blocked with 5% skim milk for 1 hour at room temperature. Then, the membranes were incubated overnight with the respective primary antibodies: anti‐USP7 (Abcam, ab190183, 1:200), anti‐ARMC5 (Abcam, ab187390, 1:100), anti‐Flag (Santa Cruz, ab205606, 1:2000), anti‐Myc (Santa Cruz, sc‐40,1:2000), anti‐PCNA (Santa Cruz, sc‐56,1:200), anti‐GAPDH (Abcam, ab8245, 1:10 000) and anti‐Ub (Abcam, ab7780, 1:5000), followed by incubation in secondary antibody for 1 hour at room temperature. The bands were detected using the ChemiDoc XRS imaging system (Bio‐Rad) using ECL detection reagents (Thermo).

Alternatively, the centrifuged protein lysates were incubated with primary antibody pre‐absorbed protein G sepharose beads at 4°C for overnight. Then, the incubation solution was washed with the RIPA buffer and boiled in 2× loading buffer for 10 minutes. Protein expression was subjected to Western blot.

### 2.3 siRNA‐USP7 and cell transfection

Negative‐control siRNA (si‐NC) and USP7‐specific siRNA (si‐USP7) were synthesized by Sangon Biotech.

USP7‐siRNA#1:5′‐UGUAUCUAUUGACUGCCCU‐3′.
USP7‐siRNA#2:5′‐CGUGGUGUCAAGGUGUACU‐3′
scrambled siRNA 5′‐UUCUCCGAACGUGUCACGU‐3′
USP7‐shRNA#1:5′‐TGTATCTATTGACTGCCCTTT‐3′
USP7‐shRNA#2:5′‐CGTGGTGTCAAGGTGTACT‐3′
ARMC5‐shRNA#1:5′‐UCUUUAGAUCGUCAUCUUGGA‐3′
ARMC5‐shRNA#2:5′‐UGUUUAAUCUCAAUUCCAGAG‐3′

Transfections were performed using Lipofectamine‐2000 transfection system (Thermo, Catalog#11668027) as described in the instruction.

### 2.4 GST pulldown assays

pGEX‐4T‐1‐USP7 was constructed according to the manufacturer's instructions. pGEX‐4T‐1‐USP7 was transfected into BL21 cells and then was induced by ispropyl b‐D‐1‐thiogalactopyranoside (IPTG). Bacterially expressed GST‐USP7 was retained on glutathione Sepharose beads (Beyotime catalog no. P2226) and incubated for 2 hours at 25°C with biosynthesis His‐ARMC5. Then, the complexes’ incubation were eluted at 100°C and subjected to Western blotting.

### 2.5 Construction of the recombinant vector and cell transfection

The full‐length USP7 open reading frame was amplified from HEK293T by RT‐PCR and cloned into the described vectors to construct the Myc‐USP7 recombinant vector. Cells were transfected with Myc‐USP7, Myc‐TUD and Myc‐TRAF using Lipofectamine 2000 (Thermo, Catalog#11668027), according to the manufacturer's instructions.

### 2.6 Protein half‐life assays

Cells were treated with cycloheximide (50 μg/mL) for various periods of time to block protein synthesis. The protein was collected and measured using Western blot.

### 2.7 In vivo ubiquitylation assay

786‐O and 769‐P cells had transfected HA‐Ub were transfected with the indicated lentiviruses or plasmid followed by treatment with 20 μmol/L MG132 for 6 hours. Cell lysates were incubated in RIPA buffer (Beyotime, catalog no.P0013B). The lysates were collected and boiled for 10 minutes. Then, cell lysates and dilution buffer (10 mmol/L Tris‐HCl, pH 8.0, 150 mmol/L NaCl, 2 mmol/L EDTA, 1% Triton) were incubated at 4°C for 30 minutes with rotation. Next, protein fraction was collected by centrifugation, followed by incubation with anti‐ARMC5 antibodies overnight. The mixture and protein A/G agarose beads were incubated for an additional 1 hour at room temperature. Then, the beads were washed three times with wash buffer (10 mmol/L Tris‐HCl, pH 8.0, 1 mol/L NaCl, 1 mmol/L EDTA, 1% Nonidet P‐40). The proteins were released from the beads by boiling beads in SDS/PAGE buffer, and they were analysed using immunoblotting with anti‐Ub monoclonal antibodies.

### 2.8 Flow cytometry assay

Cells were harvested using cell dissociation buffer (Cat#13151014, Invitrogen) and fixed in 70% ethanol at 4°C for 12 hours. Then, DNA was stained with 0.03 mg/mL propidium iodide containing RNase at 37°C for 30 minutes and subjected to analyse by flow cytometry (Beckman Coulter Life Sciences).

### 2.9 Soft agar assay

786‐O cells that had been transfected with NC‐shRNA or USP7‐shRNAs were suspended with RPMI 1640 supplemented with 0.2% agarose. Then, the above mixture was plated on the top of a solidified layer of RPMI 1640 with 0.6% agarose. The colonies were measured after three weeks. All experiments were conducted in triplicate and repeated twice.

### 2.10 The CCK‐8 assay

The cells were added to the medium counting kit‐8 reagents and incubated at 37°C for 2 hours. The absorbance was measured at the indicated time points (0, 12, 24, 48 and 72 hours). The data were analysed from three independent experiments.

## 3 RESULTS

### 3.1 ARMC5 is decreased in the renal cancer cell tissues and lines

**Figure 1:**
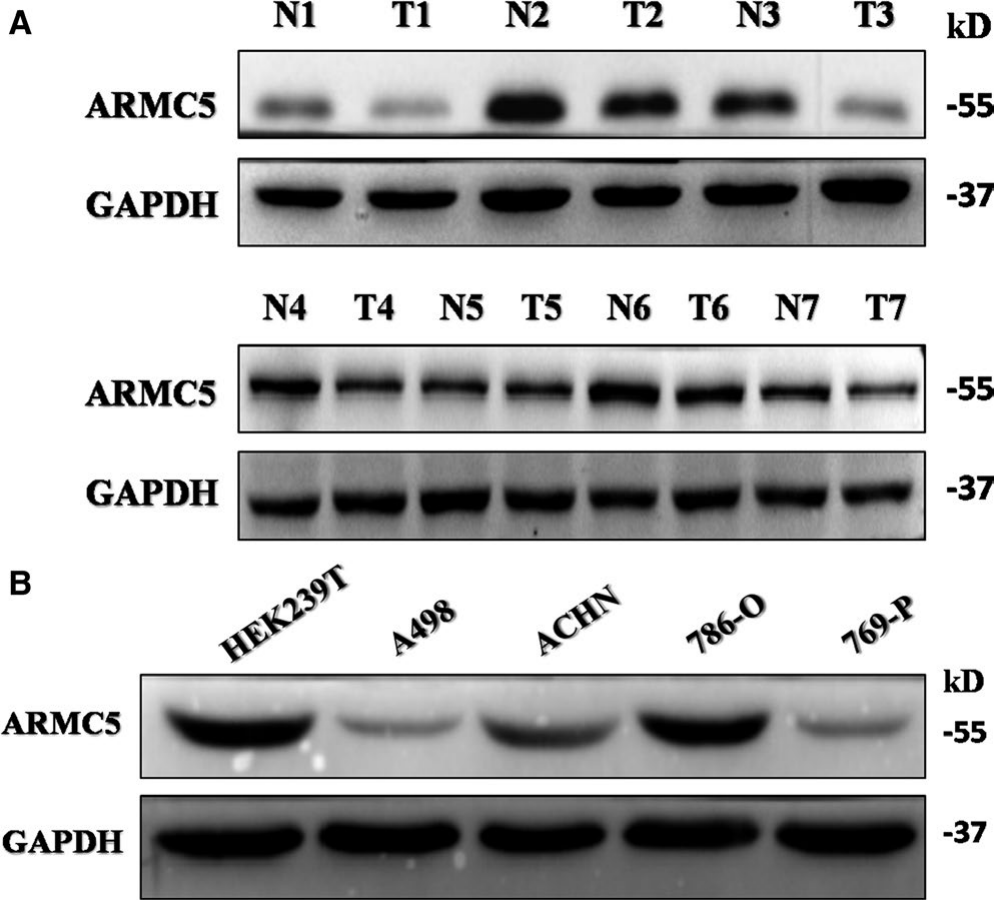
ARMC5 is decreased in the renal cancer cell tissues and lines. A, Western blot analysis of ARMC5 expression in 7 paired RCC adjacent and tumour tissues. B, Expression of ARMC5 in RCC lines and human embryonic kidney 293T (HEK293T). Western blot analysis of ARMC5 expression in the RCC cell lines. GAPDH served as an internal loading control

To assess expression levels of ARMC5 in RCC, we first measured protein levels of ARMC5 in paired non‐tumour tissue and RCC obtained from the Pathology Department. We found that ARMC5 protein levels were decreased in RCC tissues compared with adjacent normal kidney tissues by Western blot (Figure 1A). To gain a closer look at the underlying roles of ARMC5, we examined the protein expression of ARMC5 in RCC cell lines. The results showed that endogenous ARMC5 expression was decreased in RCC cell lines, compared with the normal human embryonic kidney HEK293T cells (Figure 1B). Among the above RCC cell lines, 786‐O and 769‐P cells showed the higher and lower ARMC5 expression levels, respectively. We selected the 786‐O and 769‐P cell lines for further experiments.

### 3.2 Screening of DUBs for ARMC5

**Figure 2:**
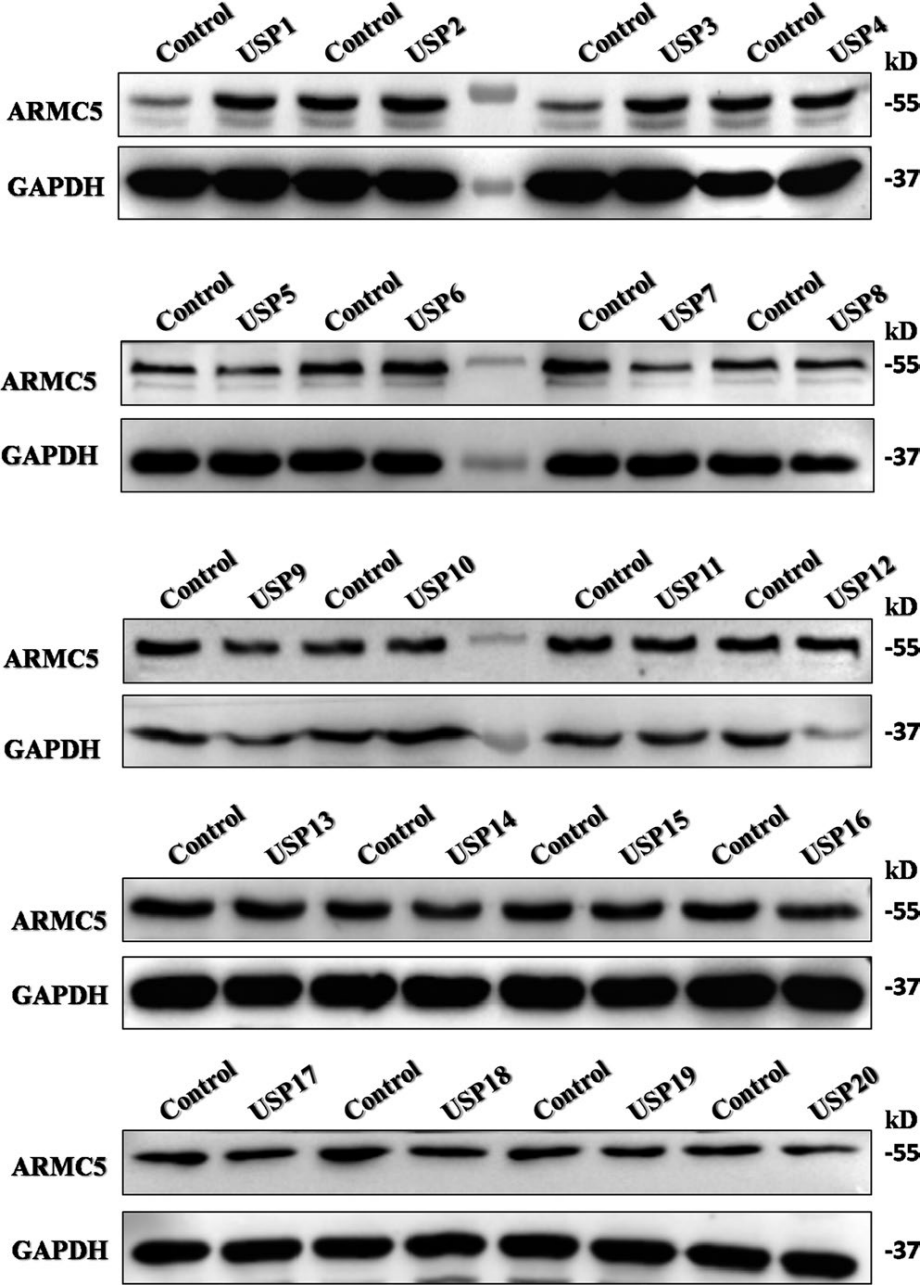
Screening of DUBs for ARMC5. The expression of ARMC 5 was measured in 786‐O cells carrying out an loss‐of‐function screen using siRNAs to inhibit 20 USPs expression

Recent studies suggest that ARMC5 interacts with E3 ubiquitin ligase CUL3, which leads to ARMC5 degradation through the ubiquitin‐proteasome pathway.^30^ Therefore, it is likely that a deubiquitinase related with ARMC5 to inhibit its degradation. To identify ARMC5‐specific DUBs, we carried out a loss‐of‐function screen to individually inhibit the expression of 20 DUBs using small interfering RNAs (siRNAs). Interestingly, we found that USP7 knockdown induced down‐regulation of ARMC5 (Figure 2).

### 3.3 USP7 interacts with ARMC5

**Figure 3:**
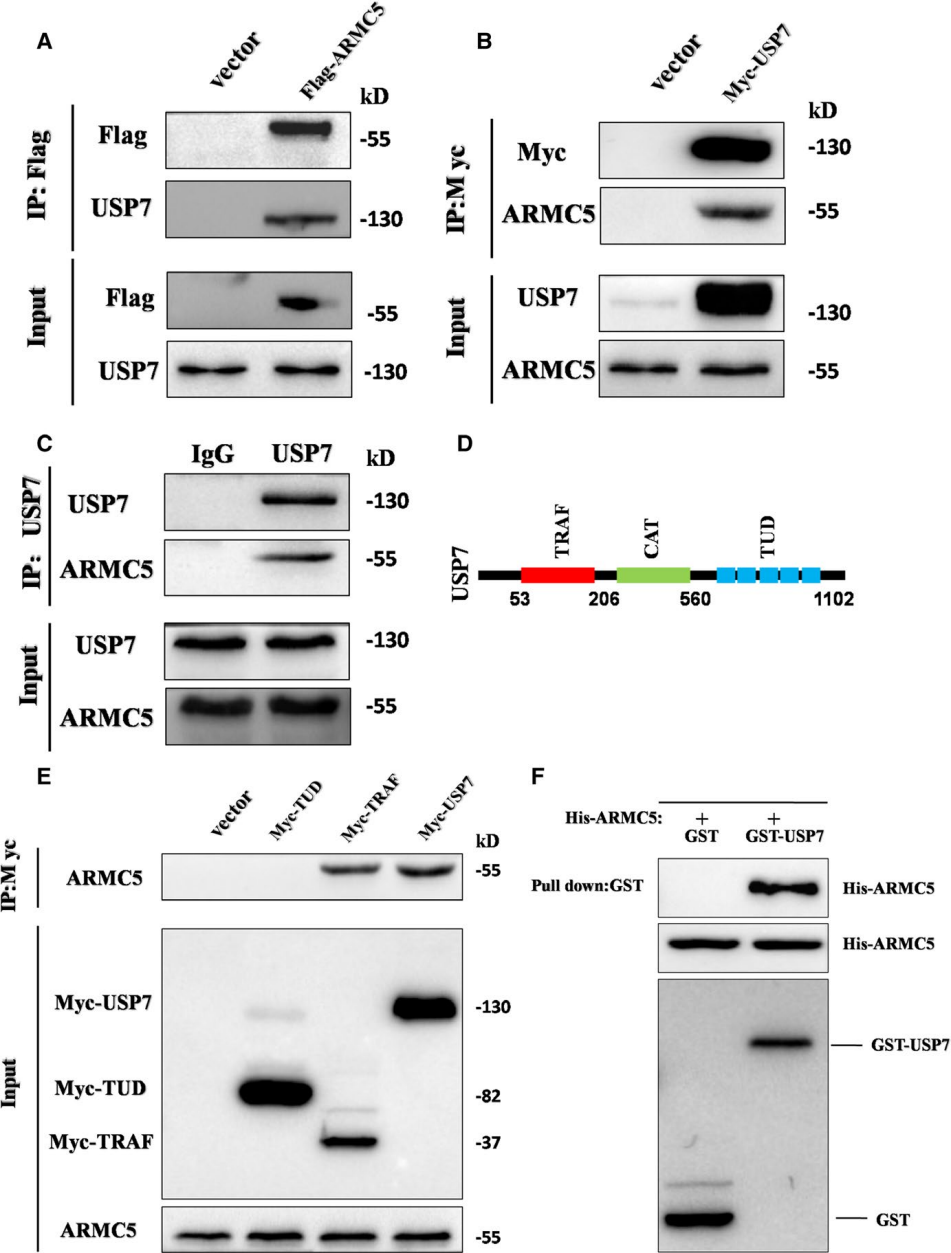
USP7 interacts with ARMC5. A, Co‐immunoprecipitation of Flag‐ARMC5 and endogenous USP7 proteins. Flag‐ARMC5 in the cell lysates from the transfected 786‐O cells were immunoprecipitated with anti‐Flag antibodies and immunoblotted with anti‐USP7 antibodies. B, Co‐immunoprecipitation of Myc‐USP7 and endogenous ARMC5 proteins. C, Co‐immunoprecipitation of endogenous USP7 and ARMC5 proteins. Endogenous USP7 in 786‐O cell lysates were immunoprecipitated with anti‐USP7 antibodies and immunoblotted with anti‐ARMC5 antibodies. D, Schematic diagram of three major domains of USP7 protein. E, Domain‐mapping analysis of which domain of USP7 mediated USP7 interacts with ARMC5. F, Recombinant GST‐USP7 pulled down recombinant ARMC5. GST and GST‐USP7 beads were co‐incubated with biosynthesis His‐ARMC5, The recombinant GST, GST‐USP7 and His‐ARMC5 proteins were detected by Western blot

As USP7 knockdown affected the levels of ARMC5, we determined whether USP7 acts directly on ARMC5. Co‐immunoprecipitation analysis revealed that endogenous USP7 was pulled down by Flag‐ ARMC5 (Figure 3A). A similar result was obtained that endogenous ARMC5 was pulled down by Myc‐USP7 (Figure 3B). The interaction between endogenous USP7 and endogenous ARMC5 was also detected by reciprocal co‐immunoprecipitations (Figure 3C). Based on the above data discussed, we next determined which domain of USP7 is responsible for ARMC5 interaction. USP7 is composed of three major domains, including an amino‐terminal tumour necrosis associated factor‐like (TRAF) domain, a middle catalytic domain and a five ubiquitin‐like folds carboxyl‐terminal tandem UBL (TUD) domain^31^ (Figure 3D). The previous research demonstrated that TRAF domain and TUD domain of USP7 are responsible for substrate recognition directly.^32^ We tested the interaction between Flag‐ARMC5 and Myc‐TRAF domain, Myc‐TUD domain of USP7 or Myc‐USP7 employing co‐immunoprecipitation assay. Domain‐mapping analysis revealed that ARMC5 was pulled down by Myc‐TRAF domain and full‐length Myc‐USP7, suggesting that USP7 bound ARMC5 through its TRAF domain (Figure 3E). To rule out the probability that interaction of USP7 with ARMC5 is through other proteins, we incubated and purified recombinant USP7 and ARMC5 in vitro. GST‐USP7, but not GST‐Control, was able to pull down His‐ARMC5 (Figure 3F). Together, these data support a direct role of USP7 in regulating ARMC5.

### 3.4 USP7 is an ARMC5 deubiquitinase

**Figure 4:**
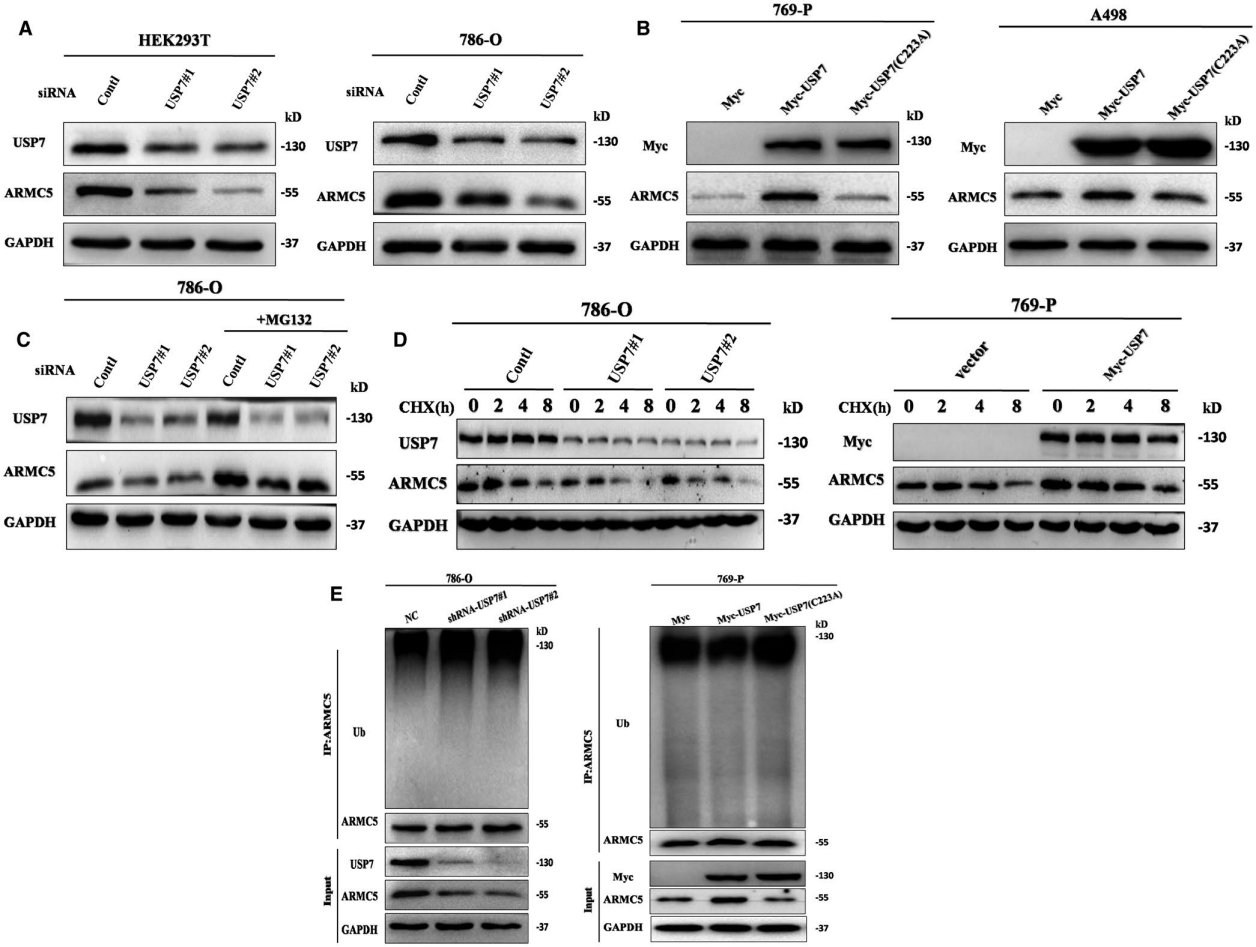
USP7 is an ARMC5 deubiquitinase. A, USP7 knockdown decreased ARMC5 protein levels. HEK293T or 786‐O cells were transfected with two independent USP7‐siRNAs. The mixed cell extracts were analysed using the Western blot. B, USP7 overexpression increased ARMC5 protein levels. Vector, recombinant Myc‐USP7 or its not catalytically mutant were transfected into 769‐P or A498 cells, and total protein was extracted and subjected to the Western blot. C, USP7 regulated ARMC5 protein levels through proteasome pathway. 786‐O cells were transfected with two independent USP7‐siRNAs. Then, cells were lysed after treatment with MG132 for 6 h. D, The half‐life of ARMC5 protein was decreased in 786‐O cells with USP7 knockdown, but its half‐life increased in 769‐P cells with USP7 overexpression. 786‐O cells transfected with the indicated siRNA‐USP7 or 769‐P cells transfected with the indicated recombinant vector were respectively treated with cycloheximide (50 μg/mL) for 0, 2,4 or 8 h. Cell lysates were then extracted and subjected to Western blotting. E, USP7 knockdown increased the levels of ubiquitinated ARMC5 and USP7 overexpression but not its mutant decreased the levels of ubiquitinated ARMC5. 786‐O cells that had transfected HA‐Ub were transfected with the indicated lentiviruses sh‐NT and sh‐USP7 or 769‐P cells had transfected HA‐Ub were transfected with vector, USP7 and its mutants followed by treatment with 20 μmol/L MG132 for 6 h, and ARMC5 from lysate was immunoprecipitated and immunoblotted with anti‐Ub antibodies to measure ubiquitination. Input displays equal protein loading

Given that silencing of USP7 decreased ARMC5 protein expression and the convinced interaction of USP7 with ARMC5, it is highly likely that USP7 serves as an ARMC5 deubiquitinase. To verify the function of USP7 as an ARMC5 deubiquitinase, we first investigated whether USP7 affects the protein levels of ARMC5. Intriguingly, silencing of USP7 performed a knockdown experiment using two independent USP7‐specific small interfering RNAs (siRNAs) in the HEK293T and 786‐O cells resulted in a significant decrease of endogenous ARMC5 levels (Figure 4A). To further confirm the regulation of ARMC5, we performed an overexpression experiment in the 769‐P and A498 cells. As shown in Figure 4B, increasing USP7 expression resulted in a significant increase of endogenous ARMC5 levels. However, the expression of ARMC5 restored to those prior to the unprocessed group when employing not catalytically USP7 mutant. Furthermore, *USP7* knockdown alone abrogated ARMC5 levels and the reduction of ARMC5 caused by USP7 knockdown could be restored by the proteasome inhibitor MG132 (Figure 4C), suggesting that USP7 maintain the protein levels of ARMC5 by abrogating its proteasomal degradation. Because the interaction of ARMC5 with USP7 and USP7 regulated the protein levels of ARMC5, we speculated USP7 was able to stabilize ARMC5. To confirm this hypothesis, cells were treated with cycloheximide (CHX) to inhibit protein synthesis in the absence of USP7 or USP7 overexpression. Protein extracts obtained at indicated time points by CHX were analysed. We found that USP7 knockdown resulted in decrease in the half‐life of ARMC5; however, overexpression USP7 led to increase in the half‐life of ARMC5 (Figure 4D). To further clarify the detailed mechanism of USP7 regulated the stability of ARMC5, we measured the levels of ARMC5 polyubiquitylation in 786‐O and 769‐P cells. The results showed that USP7 knockdown using USP7‐shRNAs led to a visible increase levels of polyubiquitylation of ARMC5. Conversely, overexpression of USP7, but not its catalytically mutant, reversed ARMC5 ubiquitination levels (Figure 4E). These data indicate that USP7 is an ARMC5 deubiquitinase.

### 3.5 USP7 alters cell cycle G1/S phases in a ARMC5‐dependent manner

**Figure 5:**
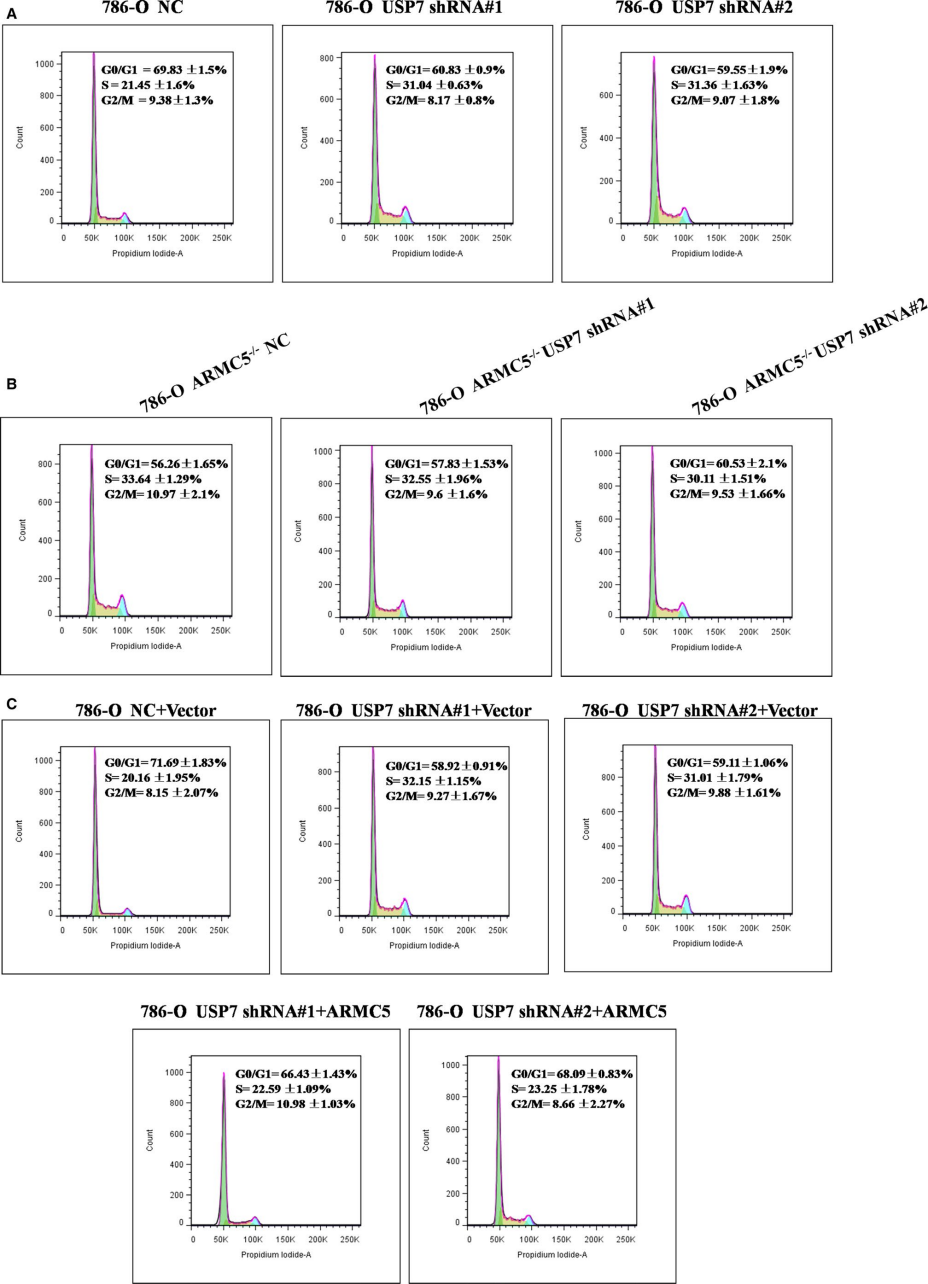
USP7 alters cell cycle G1/S phases in a ARMC5‐Dependent Manner. A, USP7 knockdown increased cell cycle G1/S phases in 786‐O cells. 786‐O cells transfected with the indicated lentiviral shRNAs‐NC or shRNAs‐USP7 were stained with propidium iodide and analysed using flow cytometry. B, USP7 knockdown exhibited no effects on the G1/S phase transition in 786‐O cells with ARMC5 was knocked down. 786‐O cells that had transfected shRNAs‐ARMC5 were transfected with the shRNAs‐NC or shRNAs‐USP7, subjected to stain with propidium iodide and analyse using flow cytometry. C, USP7 knockdown alone promoted G1/S transition, but simultaneous expression of exogenous ARMC5 reversed cell G1/S transition. 786‐O cells transfected with the lentiviral shRNAs were transfected with the indicated constructs for 24 h. Cells were stained with propidium iodide and analysed using flow cytometry

Recent research shows that ARMC5 is a cell cycle regulator, which alters G1/S phases.^30^ We hypothesized that USP7 may affect cell cycle G1/S phases. To confirm this hypothesis, the percentage of S phase cells was determined by measuring the DNA content. As predicted, the percentage of S phase with USP7 knockdown in 786‐O cells was increased compared with the control group (Figure 5A). To confirm whether USP7‐mediated G1/S transition is dependent on ARMC5, we next constructed ARMC5^−/−^ cells by ARMC5‐specific short hairpin RNA (shRNA). In contrast, USP7 depletion exhibited no effects on the G1/S phase transition in 786‐O ARMC5^−/−^cells (Figure 5B). Meantime, USP7 knockdown alone promoted G1/S transition, but silencing of USP7 cells transfected with exogenous ARMC5 could reverse G1/S transition (Figure 5C). The above results adequately suggest that USP7‐mediated G1/S transition is dependent on ARMC5.

### 3.6 USP7 regulates renal cancer cell proliferation by regulating ARMC5

**Figure 6:**
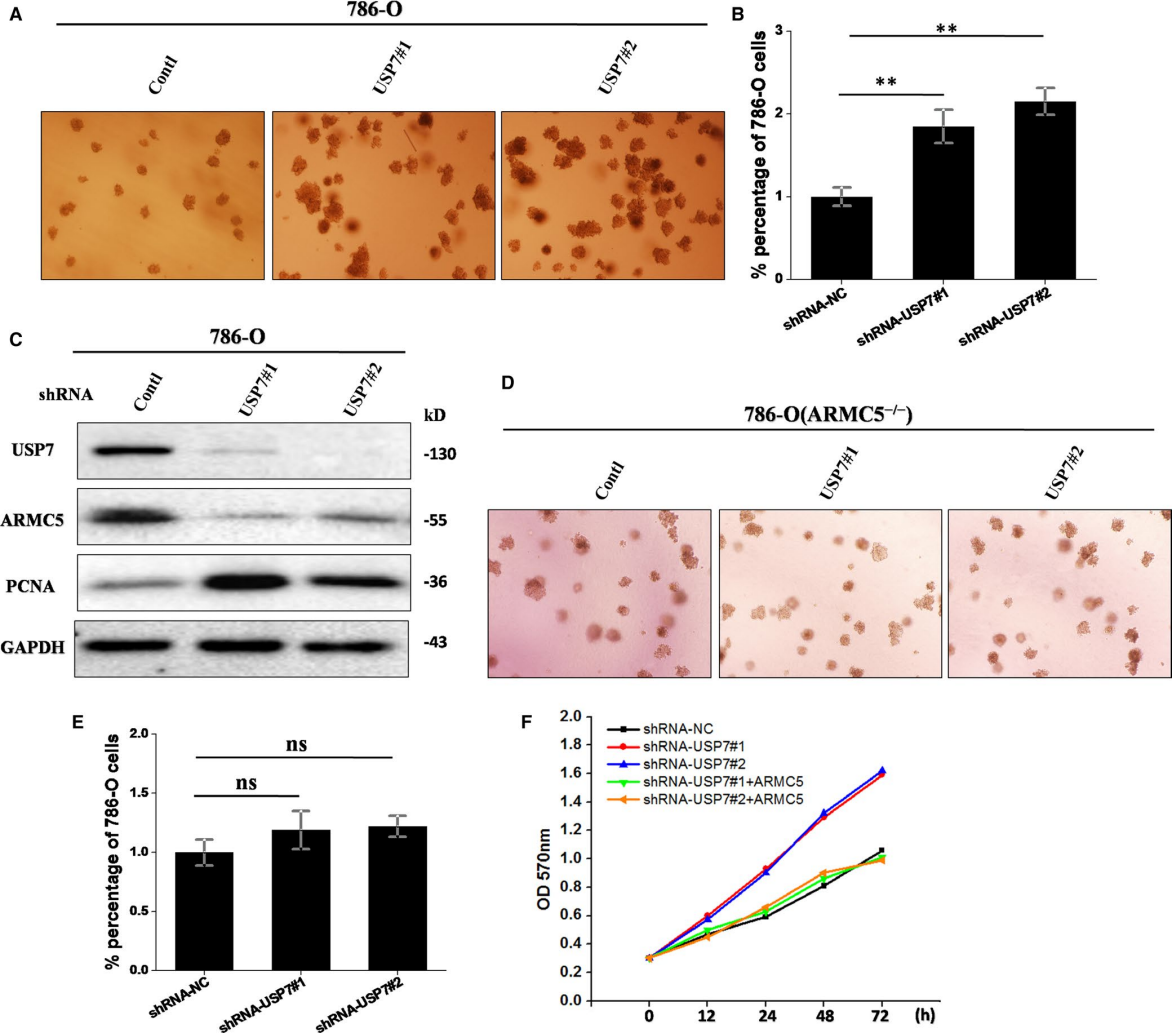
USP7 regulates renal cancer cell proliferation by regulating ARMC5. A and B, Silencing of USP7 promoted the 786‐O cells’ proliferation. 786‐O cells transfected with the indicated lentiviral shRNAs‐NC or shRNAs‐USP7 were suspended with RPMI 1640 supplemented with agarose. Three weeks later, colonies larger than 50 μmol/L were counted. The error bars indicate the mean ± SD of three independent experiments. *P < .05. C, USP7 alone knockdown increased the expression of PCNA. 786‐O cells had transfected with the indicated lentiviral shRNAs were lysed. The total protein was extracted and subjected to Western blotting. D and E, Silencing of USP7 abrogated the enhanced cell proliferation in 786‐O ARMC5^−/−^cells. 786‐O ARMC5^−/−^ cells had transfected with the indicated lentiviral shRNAs were suspended with RPMI 1640 supplemented with agarose and counted. The error bars represent the mean ± SD of three independent experiments. F, Simultaneous expression of ARMC5 prevented cell proliferation induced by USP7 knockdown. 786‐O cells transfected with the indicated shRNAs were transfected with the indicated constructs for 24 h. The above cells were added with kit‐8 reagents and incubated at 37°C for 2 h. The absorbance was measured at indicated time points. The data were analysed from three independent experiments

Given that USP7 could mediate G1/S transition by targeting ARMC5, it is likely that USP7 is participate in the regulation of cell proliferation. To determine the effect of USP7 on cell proliferation, we transfected the 786‐O cells with a control‐shRNA and USP7‐shRNA to knockdown USP7. The soft agar colony formation experiment displayed that silencing of USP7 promoted the 786‐O cells’ proliferation compared to the unprocessed group, and the difference between these two groups was, respectively, statistically significant (*P* = .0006, *P* = .0004) (Figure 6A,B). Proliferating cell nuclear antigen (PCNA), as a excellent indicator of cells proliferation, has been reported to be markers of tumour cells.^33^ To further confirm the result, we next surveyed the levels of the PCNA and found that PCNA expression was increased with USP7 alone knockdown (Figure 6C). Furthermore, silencing of USP7 in 786‐O ARMC5^−/−^cells abrogated the enhanced cell proliferation (*P* = .196) (Figure 6D,E). In addition, we found that USP7‐depleted cells transfected with exogenous ARMC5 fully prevented the cell proliferation using the CCK‐8 assay (Figure 6F). Therefore, we confirm that USP7 plays a positive role in regulating RCC cell proliferation by targeting ARMC5.

## 4 DISCUSSION

Renal cell carcinoma (RCC) is one of the leading cause of cancer‐related death in urinary system, and its incidence accounts for 3.7% in all malignant tumour.^34^ 25% patients of localized renal cell carcinoma suffer from rapid cell proliferation,^4^ which is the major causes of death among RCC patients. With the comprehensive utilization of various detection methods, there have been a few treatment options on kidney cancer during the last 2 decades.^35^, ^36^ However, RCC remains one of the most lethal urological malignancies. It is essential to reveal the molecular mechanisms of RCC proliferation to develop targeted therapy for kidney cancer. RCC originates in the renal tubular epithelial cells, and PMAH is a bilateral adrenal cortical nodules disease clinically. The current researches show ARMC5 gene mutations, including meaningless and frameshifted mutation, have been deemed to the main cause of PMAH and a relation with meningioma. Based on these findings, we hypothesized that ARMC5 might has some unique uncovered functions in RCC. In the present study, we found that the expression of ARMC5 was decreased at protein levels in the renal cancer cell tissues and lines. These results indicated that loss of ARMC5 may contribute to tumorigenesis and ARMC5 may function as a tumour suppressor in RCC.

ARMC5 is ubiquitously expressed in human tissues and encodes a protein which contains two major domains, including a N‐terminal Armadillo repeat domain and a C‐terminal BTB domain,^37^ as the docking platforms for numerous proteins. Recent studies have pointed that ARMC5 interacts with E3 ligase CUL3 through its BTB domain, leading to ARMC5 ubiquitination and further degradation by the proteasome pathway. Considering that the low expression of ARMC5 in RCC cell lines and its degradation by ubiquitination, we hypothesized that a certain enzymes function as deubiquitinase that reverses ubiquitinated ARMC5. Interestingly, we screened USP7 as an ARMC5‐associated deubiquitinase by a DUBs library. Meantime, we found that there was an interaction between USP7 and ARMC5 directly. Silencing of USP7 decreased ARMC5 levels, accompanied by increasing ARMC5 polyubiquitination, whereas overexpression of USP7 but not its mutants increased ARMC5 levels and reversed ARMC5 polyubiquitination. In summary, USP7 could stabilize ARMC5 by directly deubiquitylation. As far as we know, this is perhaps the first report demonstrating that USP7 is an ARMC5 deubiquitinase.

It has been reported that ARMC5 is participate in the regulation of cell cycle. Our results showed that USP7 altered G1/S phases in a ARMC5‐dependent manner, as illustrated that USP7 knockdown alone promoted G1/S transition, whereas silencing of USP7 cells transfected with exogenous ARMC5 reversed G1/S transition. We also confirmed that USP7 acts as tumour suppressor in RCC cells, as demonstrated that cells with USP7 knockdown exhibited increased proliferation. This is accordance with previous reports that USP7 functions as a tumour suppressor.^38^, ^39^ In contrast, silencing of USP7 had no effect on the cell proliferation in 786‐O ARMC5^−/−^ cells. Similarly, 786‐O cells with USP7‐depleted transfected with exogenous ARMC5 restored the cell proliferation, indicating that USP7 exerts its function via ARMC5.

## CONFLICT OF INTERESTS

The authors declare no conflict of interest.

## AUTHOR CONTRIBUTION

**Guobei Yan:** Data curation (equal); Formal analysis (equal); Methodology (equal); Project administration (equal); Writing‐original draft (lead). **Jun Tian:** Data curation (equal); Investigation (equal); Project administration (equal). **Na Liu:** Formal analysis (equal); Funding acquisition (equal); Investigation (equal); Project administration (equal). **Junhua Wang:** Data curation (equal); Funding acquisition (supporting); Project administration (equal); Writing‐original draft (equal). **Kai Li:** Data curation (equal); Funding acquisition (equal); Resources (equal); Software (equal); Writing‐original draft (equal). **Yanli Fu:** Investigation (equal); Methodology (equal); Project administration (equal). **Jingjing Zou:** Methodology (equal); Project administration (equal); Software (equal). **Wei Wei:** Investigation (equal); Methodology (equal); Project administration (equal). **Qing Wang:** Investigation (equal); Methodology (equal); Software (equal). **Suping Li:** Project administration (equal).

## Data Availability

Some or all data, models, or code generated or used during the study are available in a repository or online in accordance with funder data retention policies (Provide full citations that include URLs or DOIs.)
